# Type-2 diabetes epigenetic biomarkers: present status and future directions for global and Indigenous health

**DOI:** 10.3389/fmolb.2025.1502640

**Published:** 2025-04-28

**Authors:** Sarah Munns, Alex Brown, Sam Buckberry

**Affiliations:** ^1^ The Kids Research Institute Australia, Perth, WA, Australia; ^2^ National Centre for Indigenous Genomics, Australian National University, Canberra, ACT, Australia

**Keywords:** type-2 diabetes, cardiometabolic disease, DNA methylation, biomarkers, epigenetic clock, Indigenous health, Indigenous data sovereignty

## Abstract

Type-2 diabetes is a systemic condition with rising global prevalence, disproportionately affecting Indigenous communities worldwide. Recent advances in epigenomics methods, particularly in DNA methylation detection, have enabled the discovery of associations between epigenetic changes and Type-2 diabetes. In this review, we summarise DNA methylation profiling methods, and discuss how these technologies can facilitate the discovery of epigenomic biomarkers for Type-2 diabetes. We critically evaluate previous DNA methylation biomarker studies, particularly those using microarray platforms, and advocate for a shift towards sequencing-based approaches to improve genome-wide coverage. Furthermore, we emphasise the need for biomarker studies that include genetically diverse populations, especially Indigenous communities who are significantly impacted by Type-2 diabetes. We discuss research approaches and ethical considerations that can better facilitate Type-2 diabetes biomarker development to ensure that future genomics-based precision medicine efforts deliver equitable health outcomes. We propose that by addressing these gaps, future research can better capture the genetic and environmental complexities of Type-2 diabetes among populations at disproportionate levels of risk, ultimately leading to more effective diagnostic and therapeutic strategies.

## 1 Introduction

Type 2 diabetes (T2D) is a systemic cardiometabolic condition of increasing global significance. It is estimated that 6.1% of the global population (529 million people) live with diabetes mellitus, with 96% of cases attributed to T2D (GBD, 2021 Diabetes Collaborators, 2023). Increasing incidence of T2D has led to a near doubling of age-standardised prevalence estimates since 1990 (3.1%), with an anticipated future age-standardised prevalence of 9.8% expected in 2050 (GBD, 2021 Diabetes Collaborators, 2023). The global surge in T2D is driven by a transition to higher calorie diets combined with increasingly sedentary lifestyles (Chatterjee et al., 2017). Following a T2D diagnosis, if blood glucose levels are not appropriately controlled, secondary complications can develop (DeFronzo et al., 2015). These complications can be classified into macrovascular (e.g., cardiovascular disease) or microvascular (e.g., retinopathy, nephropathy and neuropathy), which significantly increases the risk of morbidity and mortality for people with T2D (Zheng et al., 2018). Of particular concern is the global increase in T2D and associated complications amongst young people, where individuals typically experience more severe clinical presentations, and have limited approved treatment options (Viner et al., 2017; Bjornstad et al., 2023). Additionally, emerging evidence indicates that young people whose mothers were living with diabetes during pregnancy have an increased risk of developing T2D and associated complications (Dabelea et al., 2008; Wicklow et al., 2018). Given the complexity of T2D aetiology and the broad range of risk factors and potential complications, T2D is a condition that would benefit from robust biomarkers that enable a precision medicine driven approach across the continuum of care.

T2D develops when insulin regulation processes break down, often following a period of partial dysfunction known as pre-diabetes, during which many patients remain asymptomatic (DeFronzo et al., 2015). This breakdown reduces the body’s ability to manage excess carbohydrate, leading to hyperinsulinaemia followed by hyperglycaemia (Roden and Shulman, 2019). To diagnose T2D, a blood screening test for either plasma glucose concentration or Haemoglobin A1C (HbA1c) levels is required (American Diabetes Association Professional Practice Committee, 2024). Due to the insidious nature of T2D, management of risks and early identification of individuals on the trajectory of T2D and complications development is vital. When people with T2D or prediabetes are identified early, lifestyle modifications through dietary and exercise changes can contribute to re-establishing blood glucose control and preventing or delaying the trajectory of T2D and associated complications for many people (Magkos et al., 2020; Siopis et al., 2021; Hocking et al., 2024). Given the effectiveness of early intervention, the development of new biomarkers that better predict T2D risk could improve early diagnosis and implementation of personalised treatment strategies.

T2D aetiology and associated complications is complex, with multiple genetic and environmental risk factors identified (Zheng et al., 2018). The 2021 Global Burden of Disease Study highlighted the impact of several factors on the overall burden of T2D, with risk factors accounting for 76.5% (95% Uncertainty Interval (UI) 58.0–87.5) of Disability Adjusted Life Years (DALYs) and of these, high body-mass index (BMI) was responsible for over half of the DALYs attributed to T2D (GBD, 2021 Diabetes Collaborators, 2023). Additionally, dietary factors played a crucial role (25.7%, 95% UI 8.6–40.7), significantly influencing the condition’s burden (GBD, 2021 Diabetes Collaborators, 2023). Environmental and occupational exposures also contributed substantially to DALYs (19.6%, 95% UI 12.7–26.5), underscoring the multifaceted nature of T2D risk (GBD, 2021 Diabetes Collaborators, 2023). Indigenous communities, particularly those impacted by settler colonialism, experience a disproportionate burden of T2D and associated complications (Harris et al., 2017). Such disparities are also observed with young people. For example, American-Indian young people (10–19 years) experience more than double the burden of T2D than American young people overall, with such disparity persisting between measurements from 2002–03 (22.6 vs. 9.0 per 100,000) and 2017-18 (46.0 vs. 17.9 per 100,000) (Wagenknecht et al., 2023). Some of the highest rates globally have been reported amongst Aboriginal and Torres Strait Islander young people (≤24 years) from northern Australia where in 2016–17, 6.7 T2D cases per 1,000 were described (Titmuss et al., 2022). Alarmingly, there are reports of Aboriginal and Torres Strait Islander children as young as 4 years old diagnosed with T2D (Titmuss et al., 2022). This younger onset T2D highlights a potential intergenerational risk, with a Canadian study observing higher incidence of T2D for people ≤30 years born to First Nations mothers experiencing T2D during pregnancy (5.63 per 1,000 person-years), compared to First Nations mothers with gestational diabetes (1.67 per 1,000 person-years) or no diabetes (0.83 per 1,000 person-years) during pregnancy (Wicklow et al., 2018). Such striking disparities and the intergenerational risk of T2D highlight an urgent need for further research into the causes and impacts of T2D in Indigenous communities. Addressing these health inequities requires targeted studies that provide the evidence needed to develop effective strategies and close the gap in T2D outcomes for Indigenous Peoples.

A meta-analysis of genomes from European (60.3%), East Asian (19.8%), ancestrally diverse African American (10.5%), ancestrally diverse Hispanic (5.9%), South Asian (3.3%) and South African (0.2%) populations revealed 611 loci associated with T2D (Suzuki et al., 2024). While a person’s genome remains largely unchanged throughout life, a multitude of environmental factors (e.g., diet, chemical exposure, chronic stress) have the potential to impact the epigenome. A cell’s epigenome comprises the modifiable biochemical molecules that interact with the genome to regulate gene expression (Portela and Esteller, 2010; Wu et al., 2023). The epigenome plays key roles in cell differentiation, development, and it influences gene expression patterns that are crucial for maintaining cellular identity and function across different tissues (Jaenisch and Bird, 2003). Epigenomics is emerging as a promising field in T2D research with multiple studies identifying epigenetic associations with T2D when profiling blood and pancreatic β-cells, as well as other tissues of relevance in T2D aetiology (Muka et al., 2016; Walaszczyk et al., 2018; Ling et al., 2022). As DNA methylation is the most widely studied epigenetic modification, this review will focus on the development of DNA methylation detection technologies and their application to T2D biomarker development. We refer to (Bannister and Kouzarides, 2011; Holoch and Moazed, 2015) for further discussion on the mechanisms of histone modifications and non-coding RNAs respectively, and (Kumar et al., 2024; Chi et al., 2021) for discussion of these epigenetic modifications in the context of T2D.

DNA methylation 5-methylcytosine (5mC), is a state where a methyl group is bound to the fifth carbon position of a cytosine base in DNA. DNA methylation commonly occurs in vertebrates at cytosines that precede a guanine base, a context known as CpG methylation (Li and Zhang, 2014). Non-CpG methylation, whilst common in plants, only typically occurs in mammals at appreciable levels in pluripotent stem cells and mature neurons (Lister et al., 2009; Luo et al., 2018; de Mendoza et al., 2021; Buckberry et al., 2023). While the intermediate demethylation product 5-hydroxymethylcytosine (5hmC) may be involved with DNA replication, transcription, cell differentiation and human disease (Kriukienė et al., 2024), discussion of 5hmC in the context of T2D biomarkers is beyond the scope of this review. Therefore, when discussing DNA methylation herein, we are referring to CpG methylation.

DNA methylation is a reversible epigenetic modification typically associated with gene repression, but is also associated with actively transcribed gene bodies and, in some contexts, with gene activation (Schübeler, 2015; Greenberg and Bourc’his, 2019). In human somatic cells, ∼70–80% of CpGs are methylated (Greenberg and Bourc’his, 2019), with significant variation between different cell types (Loyfer et al., 2023). While some transcription factors are sensitive to CpG methylation (Yin et al., 2017), which can impact gene expression, knowledge of precisely how DNA methylation regulates gene expression remains incomplete, likely with distinct functions in different genomic regions and cell types (Zhu et al., 2016; de Mendoza et al., 2022). DNA methylation is a dynamic process with methyl groups added to cytosines by DNA methyltransferase (DNMT) enzymes and removed either passively during incomplete transfer of methylation patterns during cell replication or actively by ten-eleven translocation (TET) proteins (Greenberg and Bourc’his, 2019). Through these mechanisms, it has been postulated that the dynamic nature of DNA methylation allows the methylome and therefore gene expression to respond to environmental stimuli (Martin and Fry, 2018; Cavalli and Heard, 2019; Law and Holland, 2019).

Identifying DNA methylation patterns that correlate with environmental factors is of particular interest in the development of biomarkers for chronic diseases, as many of these conditions develop due to an interplay between genetic and environmental influences (Boye et al., 2024). While genetic variants can be useful for identifying people at increased risk of developing a condition, they give little indication of current health states or reflect response to therapy. In contrast to genetics, a person’s environment can change throughout life with a myriad of exposures from diet to heavy metals, pollution, tobacco smoke and other toxins known to associate with DNA methylation levels in individual genes or larger global patterns of change (Martin and Fry, 2018; Yousefi et al., 2022). While changes to the epigenome that impact cancer susceptibility are well reported, emerging evidence indicates a role for the epigenome in T2D (Feinberg, 2018). Given the recent global surge in T2D prevalence, it is highly likely that the environmental factors that heavily contribute to T2D aetiology, likely also influence DNA methylation in some tissues. Therefore, identifying DNA methylation patterns that associate with T2D incidence and progression could contribute to the development of new predictive tools that improve our ability to identify at-risk individuals, thereby reducing the burden of this condition.

## 2 Technologies for identifying DNA methylation changes associated with T2D

The study of DNA methylation has advanced exponentially since 1975 when it was first postulated that DNA methylation contributed to gene regulation (Holliday and Pugh, 1975). Since then, a plethora of indirect and direct sequencing techniques have been developed to profile genome-wide DNA methylation patterns. This review will focus on the most commonly utilised strategies; candidate gene analysis, microarray based technologies, and next-generation sequencing (short read sequencing), as well as the emerging potential of long-read sequencing. For discussion on liquid chromatography, mass spectrometry, methylation sensitive restriction enzymes, affinity enrichment and biosensing, see (Zhang et al., 2024).

### 2.1 Candidate gene analyses

Candidate gene analyses are the screening of individual CpG sites for methylation within small regions of interest that are selected *a priori*, such as a gene promoter, typically by bisulfite PCR sequencing (Frommer et al., 1992). This technique uses sodium bisulfite to chemically convert unmethylated cytosines to uracil, leaving methylated cytosines as cytosine (Frommer et al., 1992). Multiple factors can influence the reliability of bisulfite PCR candidate gene analysis including, but not limited to, inefficient bisulfite conversion leading to an overestimation of DNA methylation (Krueger et al., 2012). While bisulfite PCR sequencing remains a popular validation method, several other methodologies have been developed, including bisulfite pyrosequencing (Pajares et al., 2021). Like bisulfite PCR, pyrosequencing utilises bisulfite conversion as the differentiating mechanism for DNA methylation, however it differs from sequencing in that differential methylation is detected via a real time luciferase reaction (Colella et al., 2003). While enabling the discovery of DNA methylation signatures in selected regions, candidate gene analyses are limited in analytical potential as they can not assess global DNA methylation patterns (Shabalin et al., 2015). However, candidate gene analysis is a cost-effective method widely used to validate microarray data.

### 2.2 DNA methylation microarrays

**FIGURE 1:**
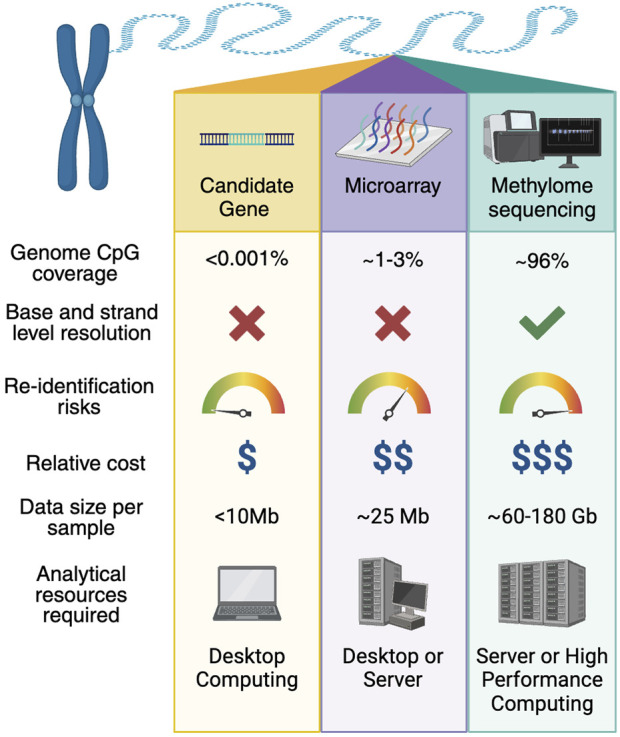
Comparison of candidate gene studies, microarrays, and methylome sequencing approaches for DNA methylation biomarker studies. Genome CpG coverage varies significantly across methods. Candidate gene approaches can typically assess tens to thousands of CpG sites, microarrays (such as Illumina 450 k and 850 k platforms) cover hundreds of thousands of CpG sites, and methylome-wide approaches capture nearly all 29.4 million CpG sites within the human genome (hg38, autosomes, X and Y). Base- and strand-level resolution highlights the ability to measure methylation at single-CpG resolution on individual DNA strands, and is only practically feasible with methylome sequencing. Microarrays provide site-level resolution based on reference genome sequence, but do not typically distinguish between strands, while candidate gene approaches are limited to specific loci typically without strand information. The potential for participant re-identification increases with the scale and resolution of the data. Sequencing-based methods pose a higher risk due to the comprehensive and individual-specific nature of the genomic data, requiring robust data governance and privacy protections. Microarrays and candidate gene studies present lower re-identification risks, as they capture less data and provide limited genomic context. Relative cost per sample reflects the resources needed for data production and analysis. Candidate gene approaches are the most cost-effective, while microarrays offer a balance of affordability, with methylome sequencing being the most costly due to sequencing and computational demands. Raw data sizes illustrate the storage demands of each method. Candidate gene studies generate minimal data (<10 MB per sample), while microarrays produce 16–20 MB per sample. In contrast, methylomes at 30x coverage produce approximately 110 GB of raw data per sample (compressed FASTQ format) and about 62 GB of mapped data (CRAM format), accounting for ∼10% data loss through PCR duplicates and read filtering, however this can be highly variable. The computational resources required increase with data complexity. Candidate gene and microarray studies can typically be processed on desktop computers or small servers, while methylome analyses often require servers or high-performance computing (HPC) environments. The shift to advanced computing infrastructure is driven by the large datasets and computationally intensive analyses associated with sequencing-based studies.

Microarray technologies capitalise on single stranded DNA’s affinity for hybridisation to a complementary strand, and underwent extensive development in the 1990s (Southern et al., 1999). One of the most widely used technologies today is the Illumina BeadChip, which uses complementary strand probes and fluorescent nucleotides to bind 50 base-pair (bp) lengths of DNA commencing at a CpG site of interest (Bibikova et al., 2009). Differential fluorescence levels then allow determination of the presence of DNA methylation in that to 50-bp lengths sequence (Bibikova et al., 2009). The CpG sites selected for screening with microarray represent only a fraction of CpG sites within the genome (Figure 1), with sequences for probes determined from the human reference genome (Noguera-Castells et al., 2023). The first large-scale DNA methylation microarray from Illumina, the 27 K BeadChip, was created in 2008 to target sequences within proximal promoter regions (Bibikova et al., 2009), but was replaced in 2011 by the 450 K microarray which expanded the scope by adding probes for a wide range of sites including but not limited to CpG islands, RefSeq gene regions, and a selection of enhancers (Bibikova et al., 2011). Microarray capacity was further expanded in 2016 with the release of the 850 K microarray, which further extended the range of enhancer sites (Pidsley et al., 2016). The latest Illumina EPIC DNA methylation microarray (900 K) includes the addition of probes to study open chromatin as well as additional enhancer locations (Noguera-Castells et al., 2023). Microarrays are widely used, particularly in large population studies, due to their lower costs and ease of use (Noguera-Castells et al., 2023). However, the limited proportion of CpG sites selected to be included on the microarray platform restricts capacity to screen for biomarkers to pre-selected regions. A recent whole genome bisulfite (WGBS) study of 205 healthy tissues representing 39 cell-types reported that the widely-used Illumina 450 K microarray and EPIC microarray only included 14% and 24% respectively of cell-type-specific differentially methylated blocks (defined as five or more CpG sites) (Loyfer et al., 2023). Microarrays also have reduced efficacy due to cross-reactivity or genomic sequence differences (Pidsley et al., 2016). As microarray development is heavily influenced by the human reference genome, current platforms under-represent global human diversity, due to the majority of the human reference genome sequence arising from one individual (Schneider et al., 2017). Thus, the suitability of microarray for measuring DNA methylation levels in individuals and populations whose genomes differ substantially from the human reference genome, is unknown.

### 2.3 Whole-genome DNA methylation profiling

With improving technology and corresponding cost reductions, sequence-based whole genome DNA methylation profiling is becoming an increasingly viable option for profiling the methylome. WGBS is a widely used method that detects methylated cytosines by fragmenting DNA, treating it with sodium bisulfite to convert unmethylated cytosines to uracil (read as thymine after PCR), and next-generation sequencing of the fragments. Methylation is determined by aligning reads to a reference genome and analysing the proportions of cytosine and thymine at CpG sites (Lister et al., 2009). The WGBS technique can produce base-level resolution coverage of ∼94% of CpG sites within the genome (Lister et al., 2009), leading to it quickly becoming the gold standard. Whilst many related enrichment-based methods coupled with sequencing exist, such as reduced representation bisulfite sequencing, they all feature biases towards different genome features or sequence contexts [reviewed in Plongthongkum et al. (2014)]. However, WGBS also features some limitations, including higher costs and the requirement of ∼200–500 ng of DNA (Li and Tollefsbol, 2021). A recently published technique that is capable of utilising existing WGBS data analysis methods is enzymatic DNA methylation sequencing (EM-Seq), which employs TET2, T4-BGT and APOBEC3A enzymes to transform methylated cytosines to uracil prior to sequencing (Vaisvila et al., 2021). EM-Seq has improved coverage of CpG rich-regions (such as promoters and CG islands), requires as little as 100 pg of DNA and gives coverage of ∼96% of 5mC bases (Vaisvila et al., 2021). Overall, the use of sequencing-based techniques capable of measuring genome-wide base-level DNA methylation levels can provide deeper insights into how DNA methylation is associated with different health states, especially when population genetic differences with respect to reference genomes may need to be accounted for.

However, the short read lengths of next-generation sequencing have a correspondingly low mapping accuracy with highly repetitive sequences (Treangen and Salzberg, 2011). These limitations can be overcome by the use of long read sequencing techniques, such as PacBio HiFi and the Oxford Nanopore sequencing, which can measure DNA methylation for long sequences of several kilobases directly from DNA, without the need for bisulfite or enzymatic conversion (Searle et al., 2023). However, these long-read technologies are not yet widely used in biomarker discovery due to the inability to rival the cost, throughput, and input DNA requirement of short read technologies (Chen et al., 2023). However, it is anticipated that long read sequencing technologies will dominate DNA methylation studies in years to come.

## 3 Improvements in DNA methylation detection technologies have accelerated the development of T2D biomarkers

### 3.1 The current state of T2D DNA methylation biomarkers

The major attraction of epigenetic biomarkers is the potential for translation to preventative and precision medicine, where improving the prediction of the likelihood of a condition would enable earlier intervention and targeted mitigation strategies (Skinner, 2024). Currently, the diagnosis of many chronic health conditions occurs at presentation of symptoms, with treatments instigated in response to diagnosis. With T2D, this occurs when the body is no longer able to cope with sustained excess glucose, with unmanaged hyperglycaemia resulting in macro and microvascular damage that can cause life threatening complications (American Diabetes Association Professional Practice Committee, 2024). If T2D is identified early, lifestyle changes (such as improving diet and exercise) and appropriate treatment, can prevent or delay T2D development (DeFronzo et al., 2015). Emerging evidence indicates DNA methylation biomarkers may contribute to improving risk stratification for T2D, with a recent longitudinal study with the Generation Scotland cohort reporting improvements in 10-year incident risk prediction scores with the addition of DNA methylation data (Cheng et al., 2023). In the following sections, we discuss the current knowledge, limitations and future opportunities in the identification of DNA methylation biomarkers for T2D.

### 3.2 Candidate gene studies

Several findings to emerge from candidate gene analyses are the identification of differential DNA methylation within the *PPARGC1A,* insulin (*INS*) and *PDX-1* gene promoters within pancreatic islets. One case-control study identified differential DNA methylation in four CpG sites in the *PPARGC1A* promoter between 10 participants with T2D (50.0% male; mean age: 65.1 years with SEM ± 2.6 years) and nine without T2D (77.8% male; mean age: 54.2 years with SEM ± 3.5 years) where the group with T2D had a higher average DNA methylation of 10.5 ± 2.7% compared to those without T2D 4.7 ± 0.9% (p < 0.04) (Ling et al., 2008). This finding extended to reduced *PPARGC1A* gene expression levels within participants with T2D (p = 0.002) (Ling et al., 2008). The study further found that knockout of *PPARGC1A* in human islets resulted in a 41% decrease (p ≤ 0.01) in insulin secretion (Ling et al., 2008). Another study investigating 25 CpG sites within the *INS* gene promoter of nine participants with T2D (55.6% male; mean age: 57.0 years with SD ± 13.1 years) and 48 participants without T2D (54.2% male; mean age: 56.7 years with SD ± 10.1 years) observed four CpG sites that had significantly increased DNA methylation in participants with T2D (Yang et al., 2011). These findings were associated with a 58% reduction (p = 0.002) in *INS* gene expression, 57% reduction (p = 0.004) in insulin content and a 26% reduction (p = 0.04) in glucose-stimulated insulin secretion (Yang et al., 2011). While investigation of the *PDX-1* gene from nine participants with T2D (55.6% male; mean age: 57.0 years with SD ± 13.1 years) and 55 participants without T2D (52.7% male; mean age: 56.7 years with SD ± 9.8 years) revealed 10 CpG sites within the distal promoter and enhancer regions that were hypermethylated (Yang et al., 2012). Expression analysis revealed *PDX-1* mRNA expression had significant positive correlation with insulin mRNA expression and glucose-stimulated insulin secretion, and negative correlation with HbA1c levels and BMI (Yang et al., 2012). Participants with T2D were observed to have significantly reduced expression of *PDX-1* (0.40 ± 0.076 compared to 1.29 ± 0.15; p = 2 × 10^−4^ for participants without T2D) (Yang et al., 2012). Further analysis within the above studies suggested that *PPARGC1A, INS*, and *PDX-1* have an important role in insulin secretion and that gene expression differences influenced by methylation patterns of these genes in T2D is associated with regulation of insulin secretion and insulin content. Moreover, preliminary data from blood samples indicate associations with reduced DNA methylation levels and increased expression for genes in insulin signalling and metabolism with T2D in chronic kidney disease (Khurana et al., 2023). Together, these studies demonstrate the ability of candidate gene analysis to detect differential DNA methylation associated with T2D for relevant genes selected *a priori*, however candidate gene analysis is typically now only utilised as a validation tool.

### 3.3 Microarray-based studies

DNA methylation microarrays have become widely used for T2D biomarker development due to their cost effective ability to screen orders of magnitude more CpG sites than candidate approaches. This has resulted in differential DNA methylation signatures being identified in clinically significant tissues for T2D (adipose, skeletal muscle, liver and pancreas), however the predominant tissue utilised for screening thus far remains blood (Muka et al., 2016; Walaszczyk et al., 2018; Willmer et al., 2018). In adipose tissue, a study which included 28 participants with T2D (53.6% male; mean age: 74.5 years with SD ± 4.2 years), age and sex matched to 28 participants without T2D, observed 15,627 differentially methylated loci within 7,046 genes with the Illumina 450 K Beadchip microarray (Nilsson et al., 2014). A KEGG pathway analysis of these genes showed significant enrichment in pathways including inflammation and glycan metabolism (Nilsson et al., 2014). While a study of skeletal muscle and subcutaneous adipose tissue using the Illumina 27 K microarray with 12 monozygotic twin pairs (50% male; mean age: 68.3 years with SD ± 7.7 years) discordant for T2D from Denmark, found that within the 11 pairs that provided skeletal muscle, CpG sites linked with *IL8* were significantly different, and within the 5 pairs that provided subcutaneous adipose tissue, CpG sites from *ZNF668, HSPA2, C8orf31, CD320, SFT2D3, TWIST1,* and *MYo5A* showed statistically significant differential DNA methylation (Ribel-Madsen et al., 2012). In a study of liver tissue using the Illumina 450 K Beadchip, significant hypomethylation at a CpG site within *PDGFA* was identified in a European ancestry cohort of 96 women with obesity and T2D (mean age: 48.2 years with SD ± 6.34 years) and 96 age and BMI matched women with obesity and without T2D (41.3% methylation for participants with T2D versus 60.3% methylation for participants without T2D) (Abderrahmani et al., 2018). These findings were replicated in a German cohort of 12 participants with T2D and 53 participants without T2D (Abderrahmani et al., 2018). Increased expression of *PDGFA* was observed to correlate with hepatic fibrosis risk, hyperinsulineamia and insulin resistance, with levels of CpG methylation at this loci also having an inverse correlation with *PDGFA* expression (Abderrahmani et al., 2018). An investigation of pancreatic islet cells from people with and without T2D identified 5,584 differentially methylated CpG sites by EPIC Beadchips that were also associated with HbA1c (Rönn et al., 2023). Intriguingly, when profiling islets from individuals not previously diagnosed with T2D, the results indicate that HbA1c-associated CpG loci are predictive of future T2D (Rönn et al., 2023). Gene expression analysis further revealed 65 differentially expressed genes that were linked to 113 CpG sites associated with T2D and HbA1c (Rönn et al., 2023). Further analysis of blood samples (collected prior to T2D diagnosis) in a longitudinal cohort revealed four sites within *NKX6.2, SYNPO, RHOT1*, and *CABLES1* that were differentially methylated (Rönn et al., 2023). Through the use of siRNA gene silencing in pancreatic islet cells, *FOXP1, TBC1D4, RHOT1* and *CABLES1* were observed to have functional involvement in glucose-stimulated insulin secretion (Rönn et al., 2023). Further analysis in *Rhot1* knock-out rat *β*-cells observed *Rhot1* was integral for glucose-stimulated insulin secretion and mitochondrial function (Rönn et al., 2023). Although some evidence of differential DNA methylation has been observed in these tissues, a common limitation of many tissue-based studies is their relatively small sample size compared to other biomarker discovery studies. This limits statistical power and increases the risks of false-negative findings. Additionally, while detectable statistical differences in DNA methylation may offer mechanistic insights for tissue-based studies, the need for tissue sampling complicates the translation of these findings into clinical biomarkers. Aside from the blood sample sub-analysis undertaken by Rönn et al. (2023), all of the above described studies have used a retrospective case-control design. Retrospective case-control studies are quicker and cheaper to undertake comparative to longitudinal studies investigating incident cases, however a major limitation in T2D biomarker research is that it is practically impossible to determine if the identified biological variation pre-dated the onset of T2D or was a consequence of T2D. By conducting longitudinal studies where samples are available prior to clinically identifiable T2D, for participants who later develop T2D, it can be possible to identify potential DNA methylation biomarkers predictive of future T2D development. See Table 1 for studies reviewed herein that have longitudinal designs.

**TABLE 1:** Summary of DNA methylation-based studies investigating Type-2 diabetes incidence.

Reference	Study size	Population summary	Study design	Main results summary
Chambers et al. (2015)	Discovery cohort: n = 1,074 with T2D (67.3% male), n = 1,590 without T2D (68.2% male) (age and sex matched) Replication cohort: n = 377 with T2D (63.9% male), n = 764 without T2D (68.3% male) (age and sex matched) Liver association: n = 175 with obesity, a total of 2,201 blood samples and 116 liver samples were available for assessment	Incidence discovery cohort: Male and female people of Indian Asian descent from the LOLIPOP study. (Age range not described, mean for participants with T2D 52.5 years with SD ± 10.2 years, mean for participants without T2D 49.9 years with SD ± 9.8 years) Replication cohort: Male and female people of European descent from the LOLIPOP and KORA studies. (Age range not described, LOLIPOP group means: participants with T2D 60.7 years with SD ± 8.7 years and participants without T2D 60.4 years with SD ± 9.7 years. KORA group means: participants with T2D 57.8 years with SD ± 8.9 years and participants without T2D 57.6 years with SD ± 8.9 years) Liver association: People of European descent	Discovery cohort: A nested incident case-control. Blood-samples collected at baseline. Study follow-up period was 8-years. DNA methylation measured by Illumina HumanMethylation 450 K microarray. 466,186 probes tested. Validation done by assessment of the top findings in the replication cohort, which is an incident case-control design with blood-samples collected at baseline. DNA methylation measured by pyrosequencing with LOLIPOP participants and 450 K microarray with KORA participants. Liver association: in paired blood and liver samples. DNA methylation measured by HumanMethylation 450 K microarray.	After replication screening 5 CpG sites associated with T2D (p < 0.05). For these sites relative risk scores for each 1% increase in methylation were: *ABCG1* (cg06500161) 1.09 (95% CI 1 · 07–1·11; p = 1·3 × 10–^1^⁷), *PHOSPHO1* (cg02650017) 0·94 (0·92–0·95; p = 4·2 × 10^–11^), *SOCS3* (cg18181703) 0·94 (0·92–0·96; p = 1·4 × 10–⁹), *SREBF1* (cg11024682) 1·07 (1·04–1·09; p = 2·1× 10–^1^⁰) and *TXNIP* (cg19693031) 0·92 (0·90–0·94; p = 1·2 × 10–^1^⁷). A T2D combined results methylation score comparing the RR of quartile 1 and quartile 4 produced a score of 3.51 (95% CI 2.79-4.42; = 1·3 × 10–^2^⁶) Liver association study observed association on *TXNIP* (p = 0.02) and *SOCS3* (p = 5.3 × 10^−5^)
Dayeh et al. (2016)	Prospective cohort: n = 129 with T2D (49.6% female), n = 129 without T2D (51.9% female) (age and gender matched) Case-control analysis: Monozygotic twin pairs discordant with T2D: n = 14 pairs provided adipose tissue, n = 17 pairs provided skeletal muscle, n = 19 pairs provided blood (note n = 9 pairs provided all three tissues) Pancreatic islet: n = 15 with T2D, n = 34 without T2D. Liver: n = 35 with T2D, n = 60 without T2D	Prospective discovery cohort: Male and female participants from the Botnia cohort recruited from Botnia, Finland. (Age range not described, group means at baseline: participants with T2D 52.8 years with SD ± 12.3 years and participants without T2D 51.4 years with SD ± 9.1 years). Case-control cohort data sourced from other investigations	To attempt to replicate the findings of the 5 CpG sites in Chambers et al. (2015) in prospectively collected blood samples. Study follow-up period was 8.1 ± 3.7 years. DNA methylation measured by pyrosequencing. Followed by analysis of these sites in a case-control study using adipose tissue, skeletal muscle and blood from monozygotic twins discordant with T2D and pancreatic islets and liver from donors with T2D and without T2D. DNA methylation measured by Infinium Human- Methylation450 BeadChips (Illumina).	In the prospective cohort *ABCG1* methylation displayed an increased risk for T2D (OR = 1.09, 95% CI = 1.02–1.16, p-value = 0.007,Q-value 0.018); *PHOSPHO1* methylation demonstrated reduced risk for T2D (OR = 0.85, 95% CI = 0.75–0.95, p-value = 0.006,Q-value = 0.018); *SREBF1, SOCS3* and *TXNIP* were all non-significant. Several significant correlations: *ABCG1* with BMI, HbA1c, fasting insulin, and triglycerides; *PHOSPHO1* with HDL; SOCS with age and BMI; *SREBF1* with age, BMI, fasting glucose and HbA1c; *TXNIP* with triglycerides. In matched blood and adipose tissue samples DNA methylation correlated for *SOCS3* and *SREBF1* (Regression coefficient = 0.31 and 0.040, p-value = 0.010 and 0.052, n = 28)
Jeon et al. (2017)	Discovery cohort - Subgroup investigating high glucose response (ΔhiGlu60) : n = 8 with ΔhiGlu60 (50% male) and n = 8 without ΔhiGlu60 (50% male) - Subgroup investigating T2D: n = 5 with T2D (100% male) and n = 5 without T2D (100% male) (age and sex matched) Replication cohort 1: n = 220 with T2D (53.6% male) and n = 220 without T2D (53.6% male) Replication cohort 2: n = 2 with T2D (100% male), n = 16 without T2D (37.5% male)	Incidence discovery cohort: Male and female participants from the KoGES study. (Age range not described, but means within the subgroup investigating T2D were 49 years with SD ± 2.68 years for both participants with T2D and without, within the subgroup investigating ΔhiGlu60 52.5 years with SD ± 4.85 years for participants with ΔhiGlu60 and 51 years with SD ± 2.24 years for participants without ΔhiGlu60) Replication cohort 1: Male and female participants from the KoGES study (Age range not described, mean of participants with T2D was 60.12 years with SD ± 7.99 years and mean of participants without T2D was 61.14 years with SD ± 9.34 years) Replication cohort 2: Male and female people that underwent pancreatectomy at Asan Medical Centers in Seoul, Korea. (Age range not described, mean was 55 ± 16 years)	Discovery cohort: An incident case-control investigation of impacts of hyperglycaemia in people with T2D and high glucose response (ΔhiGlu60). Diabetes diagnosis occurred at 10 years follow-up (5th phase). Blood samples collected at baseline and follow-up. DNA methylation measured by Infinium Human Methylation 450 K Bead microarray. Validation in replication cohorts. Replication cohort 1: Blood samples from participants with T2D or without T2D at the 5th phase follow-up pyrosequenced at chr17:55484635 site of the *MSI2* gene. Note: pyrosequencing of the *MSI2* cg23586172 site (chr17:55484600) identified in discovery was reported to not be possible, so the nearest site was selected. Replication cohort 2: Screening of methylation by WGBS in pancreatic islets at the chr17:55484635 site (hg19) and then ± 5 kbs either side	Discovery cohort: 153 differentially methylated sites identified in the subgroup of participants investigating T2D and 229 sites identified within the subgroup of participants investigating ΔhiGlu60, three of which were common between subgroups: *MSI2* (cg23586172), *CXXC4* (cg22604213) and unnamed intergenic (cg25290098). The *MSI2* site was hypomethylated by 11% (p-value = 0.0038) and 7% (p-value = 0.038) in the subgroups investigating T2D and ΔhiGlu60 respectively. The *CXXC4* site was hypomethylated by 15% (p-value = 0.044) and 12.8% (p-value = 0.033) in the subgroups investigating T2D and ΔhiGlu60 respectively. Replication cohort 1: revealed a hypomethylation association with T2D (p-value = 2.20 × 10^-16^), and a mean decrease in methylation of 3%. Replication cohort 2: found 72% methylation in islets from people without T2D, while 56% methylation was documented in islets from people with T2D at the chr17:55484635 site. On WGBS sequencing assessment 39 statistically significant DMPs were identified. See Table 2 for details.
Cardona et al. (2019)	Discovery cohort: n = 563 with T2D (84% female), n = 701 without T2D (58% female) Confirmation cohort 1: n = 1,074 with T2D (36.3% female), n = 1,590 without T2D (31.8% female) (age and sex matched) Confirmation cohort 2: n = 403 with T2D (43.0% female), n = 2,204 without T2D (56.5% female)	Incidence discovery cohort: Male and female people of European descent from England and Wales enrolled in EPIC-Norfolk. (Age range not described, but mean is 61.6 years with SD ± 8.1 years for participants with T2D, and 59.1 years with SD ± 9.2 years for participants without T2D). Confirmation cohort 1 (incidence): Male and female participants of Indian Asian descent from the LOLIPOP study. (Age range not described, but mean is 52.5 years with SD ± 10.2 years for participants with T2D, and 49.9 years with SD ± 9.8 years for participants without T2D). Confirmation cohort 2 (prevalence): Male and female participants from the Offspring cohort of the Framingham Heart Study (FHS). The original FHS cohort are people of European descent from Framingham. (Age range not described, but mean is 69.3 years with SD ± 8.4 years for participants with T2D, and 65.8 years with SD ± 8.9 years for participants without T2D)	Discovery cohort: A nested incidence case-cohort study drawn from the wider EPIC-Norfolk study. Blood-samples collected at baseline. For participants with T2D, this was up to 11 years prior to diagnosis. DNA methylation measured by Human Methylation 450 K BeadChip, with 442,920 probes per sample after QC. Confirmation cohort 1: A nested incident case-control study drawn from the wider LOLIPOP study. Blood-samples collected at baseline. DNA methylation measured by Human Methylation 450 K BeadChip. Confirmation cohort 2: A prevalent case-control study. Blood samples collected on the eighth study visit. DNA methylation measured by Human Methylation 450 K BeadChip, 443,304 probes per sample after QC. Tested against externally sourced Illumina Infinium Human Methylation 450 K BeadChip data from blood, liver, adipose tissue and skeletal muscle Underlying methylation quantitative trait loci (meQTL) for identified CpG loci were then screened on Mendelian randomisation	Three previously identified (cg19693,031[*TXNIP*], cg06500161 [ *ABCG1*], cg11024682 [ *SREBF1*]) and 15 novel (cg14476101 [*PHGDH*], cg14020176 [*SLC9A3R1*], cg06397161[*SYNGR1*], cg00574958 [*CPT1A*], cg06235429 [*NDUFV1*], cg05778424 [*AKAP1*], cg11376147 [*SLC43A1*], cg04816311 [*C7orf50*], cg02711608 [*SLC1A5*], cg08309687, cg13514042, cg08994060 [*PFKFB3*], cg01676795 [*POR*], cg25130381 [*SLC9A1*], cg11183227 [*MAN2A2*]) CpG loci identified in the discovery cohort. In the confirmation cohorts: 14/18 (p < 0.05) were methylated consistently in cohort 1 (incidence), 16/18 (p < 0.05) were methylated consistently in cohort 2 (prevalence). However when the 18 CpG sites were assessed in aggregate in confirmation cohort 1 (incidence) no prediction capability was observed. meQTL via Mendelian randomisation identified cg00574958 [*CPT1A*] as causally associated with T2D (P = 0.01)
Wittenbecher et al. (2019)	Subset of the cohort that provided samples for use in the DNA methylation investigation: n = 300 with T2D and n = 300 without T2D (age, sex, fasting time, and blood draw season and time matched).	Male and female participants of European descent from the EPIC-Potsdam cohort	Nested case-cohort incident study screening blood insulin-like growth factor protein 2 (IGFBP-2) concentrations Blood samples collected at baseline (recruitment period 1994–1998). Last date for follow-up 31st August 2005. DNA methylation analysis undertaken on this nested subset of participants. DNA methylation detected by Infinium MethylationEPIC BeadChip returning 890,703 probes. 33 CpG sites were within the *IGFBP-2* gene.	7 CpG sites within *IGFBP-2* had a statistically significant risk correlation after multiple testing correction
Huang et al. (2021)	n = 287 with T2D and n = 287 without T2D (sex, age, marital status, ethnicity, and residence village matched) (65.51% female)	Male and female participants enrolled in the Rural Chinese Cohort Study (Age range not described, mean age = 52.27 ± 9.53 years, at baseline)	Nested case-control incident study of 19 CpG sites within the promoter region of the *FTO* gene (Chr16: 53703509–53703936). 6 years follow-up period Blood-samples collected at baseline. DNA methylation detected by MassARRAY EpiTYPER	CpG9 within the *FTO* gene showed significant correlation with T2D OR 2.19 (95%CI: 1.31–3.65)
Qie et al. (2021)	n = 286 with T2D and n = 286 without T2D (sex, age, marital status, race, and residence village matched) (66.08% female)	Male and female participants enrolled in the Rural Chinese Cohort Study from Xin’an, China (median age = 53 years, IQR 45-49 at baseline)	Nested case-control incident study of 15 CpG sites within chr21:43656137–43657036 of the *ABCG1* gene (one CpG (CpG13) was previously described (cg06500161) and one was undetected). 6 years follow-up period Blood-samples collected at baseline and follow-up. DNA methylation detected by MassARRAY EpiTYPER.	At baseline: No significant associations observed in the unadjusted model. Post-adjustment, for every 1% methylation increase in CpG 13 and CpG 14 a 16% increase in risk was observed (OR = 1.16, 95% CI = 1.02–1.31) Across the study period: CpG15 had significant DNA methylation change (p = 0.010). Post adjustment those with greater methylation gain (≥5%) compared to lower gain (<1%) at CpG15 had increased risk (OR = 1.78, 95% CI = 1.01–3.15)
Domingo-Relloso et al. (2022)	n = 348 with T2D (39.7% male) n = 964 without T2D (45.5% male)	Male and female participants of American Indian descent (Median age 52.9 years with IQR 48.4, 60.2 years for participants with T2D and median age 54.3 years with IQR 48.7, 61.2 years for participants without T2D)	Prospective. Blood samples collected at baseline (1989–1991) with follow-up assessments 1993-1995 and 1998-1999. DNA methylation measured by Illumina’s MethylationEPIC BeadChip (850 K) producing 788,368 probes.	49 differentially methylated positions were identified, post multiple comparisons correction none significant
Fraszczyk et al. (2022b)	n = 132 with T2D (45% female) n = 132 without T2D (45% female) (age and sex matched)	Male and female participants from the prospective Doetinchem Cohort Study, Netherlands (Age range not described. Mean age of participants with T2D = 60.1 years at time of T2D diagnosis, mean age of participants without T2D = 60.0 years)	A nested case-control incidence study investigating 107 CpG sites previously associated with incident and prevalent T2D. Blood samples collected at time of T2D diagnosis and ∼ five and ∼10 years prior. DNA methylation measured by Illumina Infinium Methylation EPIC chip producing 803,591 probe results per sample after QC	Over the three timepoints 10 CpGs showed variation in slope between participants with T2D and participants without T2D, four significant at two or more time points: cg06500161 (*ABCG1*), cg08994060 (*PFKFB3*), cg15020801 (*PNPO*), and cg19693,031 (*TXNIP*); Eight had parallel variations across all three timepoints with cg11024682 (*SREBF1*), cg11202345 (*LGALS3BP*), cg05778424 (*AKAP1*), cg19750657 (*UFM1*), and cg07504977 continually hypermethylated and cg14476101 (*PHGDH*), cg18181703 (*SOCS3*), and cg26262157 (*PFKFB3*) continually hypomethylated. Strong correlation with chronological age seen with GrimAge, Hannum, Horvath and PhenoAge clocks; Participants with T2D had higher age estimates than participants without T2D on all clocks, but this was not statistically significant. GrimAge predicted participants with T2D and participants without T2D to have biological age older than chronological age. Other 3 had participants with T2D and participants without T2D with lower biological ages
Fraszczyk et al. (2022a)	Discovery cohorts: n = 1,250 with T2D, n = 1,950 without T2D (males comprised 42%–68.1% across the cohorts) (mixture of age, sex and measurement matched, and non-matched) Replication cohort: n = 1,072 with T2D (67.3% male), n = 1,587 without T2D (68.2% male) (age and sex matched)	Discovery cohorts (prospective): Male and female participants from five prospective cohorts (Doetinchem Cohort Study, Netherlands; ESTHER study, Germany; KORA1, Germany; KORA2, Germany; EPIC-Norfolk, UK) of people of European descent. (Age range not described, but mean across the cohorts were 50.3–62.7 years) Replication cohort (incidence): Male and female people of Indian Asian descent from the LOLIPOP study (Age range not described, participants with T2D mean 52.6 years with SD ± 10.2 years, participants without T2D mean 49.9 years with SD ± 9.8 years)	A meta-analysis of the 5 prospective cohorts that had follow up times of 6.25–10.5 years. DNA methylation was measured from blood by Illumina Infinium Methylation EPIC chip (Doetinchem cohort) and Illumina Infinium Human Methylation 450K (remaining cohorts). Number of probes utilised ranged from 416,716 - 470,870. Replication cohort: Significant findings from the five cohorts were tested in the LOLIPOP cohort. DNA methylation was measured from blood by Illumina Infinium Human Methylation 450K. Number of probes 466,186.	76 differentially methylated CpG sites in the discovery cohort (10 most significant sites annotated to *TXNIP, PHGDH, C7orf50, CPT1A, OLMALINC, MAP4K2, UFM1,* *SREBFq, AKAP1,* and *ABCG1*), 64 of which correlated in the replication cohort. After adjusting for baseline BMI 4 CpG sites (*TXNIP, ABCG1, CFL2,* and *TRIO*) significant
Hillary et al. (2023) ^a^	Prevalence participants: n = 348 with T2D, n = 14,002 without T2D Incidence participants: n = 534 with T2D, n = 13,437 without T2D (T2D gender breakdown not listed, but 58.8% of the cohort were female) (corrected for age and sex)	Male and female participants of European ancestry from the Generation Scotland (GS) family cohort study who were recruited to investigate 14 prevalent diseases and 19 incident conditions. T2D was considered both prevalently and incidentally. (Age distribution of participants in the substudy investigating T2D was not listed. For whole GS cohort 18–99 years, mean 47.5 years with SD ± 14.9 years)	Participants were recruited in 2006–2011 with health record screening in October 2020 to identify people that had developed T2D since baseline (incidence). Blood samples were collected at baseline with DNA methylation measured by Illumina Infinium MethylationEPIC microarray with 752,722 probes after QC.	Prevalence investigation 52 CpG sites were significantly associated with T2D. CpG sites with the top 5 smallest p-values cg19693,031 (*TXNIP*), cg06500161 (*ABCG1*), cg17901584 (*DHCR24*), cg15659943 (*ABCA1*), cg02988288 (*TXNIP*). 10 CpG sites have been reported previously Incidence investigation 58 CpG sites were significantly associated with T2D. CpG sites with the top 5 smallest p-values cg19693031 (*TXNIP*), cg06500161 (*ABCG1*), cg27243685 (*ABCG1*), cg11024682 (*SREBF1*), cg00163198 (*SNX19*). 8 CpG sites have been reported previously. 17 CpGs mapping to 11 genes were common between prevalence and incidence investigation^a^
Rönn et al. (2023)	Prevalence cohort -Subcohort 1 investigating T2D study: n = 25 with T2D (68.0% male), n = 75 without T2D (61.3% male) -Subcohort 2 investigating HbA1c: n = 114 without T2D (64.0% male). Note: some participants shared with subcohort 1. Total 139 islets across both cohorts Validation cohorts: Dayeh et al. (2014) data n = 15 with T2D, n = 34 without T2D (note: 5 with T2D and 18 without T2D were also participants in the prevalence cohort of this study). Volkov et al. (2017) data n = 6 with T2D and n = 8 without T2D) Incidence cohort: n = 270 with T2D (51.9% male), n = 270 without T2D (51.9% male) (matched for age, sex, time of fasting, time of blood draw, and season of sample collection)	Prevalence cohort: Male and female participants from the Nordic Network for Islet Transplantation in Uppsala, Sweden. (participants with T2D were 45–81 years, mean 62.8 years; participants without T2D 43–81 years, mean 61.4 years; participants in the subcohort investigating HbA1c 24–81 years, mean 58.8 years) Validation cohorts: Male and female participants from previous pancreatic islet case-control studies by Dayeh et al. (2014), Volkov et al. (2017) Incidence cohort: Male and female participants from the EPIC-Potsdam study (Age range not described, mean 54.4 years with SD ± 7.5 years)	Prevalence cohort: A case-control study assessing for methylation variation in both HbA1c and T2D contexts within pancreatic islets. DNA methylation was measured by Infinium MethylationEPIC BeadChips with 816,888 probes. RNA-Seq data from 25 participants with T2D and 72 without T2D was also collected. Pyrosequencing on four sites in genes *CDKN1A, HDAC4, TXNIP*, and *RHOT1*, using 24 samples from participants with T2D and 59 samples from participants without T2D from the investigating T2D subcohort. Validation cohorts: Findings then compared to data from case-control studies with pancreatic islets. Dayeh et al. (2014) measured DNA methylation with 450K microarray and Volkov et al. (2017) measured DNA methylation with WGBS. Incidence cohort: A nested case-control study with a median T2D diagnosis time of 3.8 years (IQR 2.0–5.3). Blood samples collected at baseline	Prevalence cohort: In the subcohort investigating T2D 31,806 sites were associated with T2D (77% were in hypomethylation) and post adjustment for cell-type heterogeneity 24,546 retained association (p = 4.2 × 10^−20^ – 4.9 × 10^−2^). In the subcohort investigating HbA1c 18,422 sites were associated and post adjustment for cell-type heterogeneity 10,938 retained association (p = 8.6 × 10^−13^ – 4.9 × 10^−2^). 5,584 sites overlapped between the sub cohorts and were directionally agreeable, although strength of difference was greater in the subcohort investigating T2D. Pyrosequencing of *CDKN1A, HDAC4*, *TXNIP*, and *RHOT1* was consistent with EPIC Beadchip data Validation cohorts: Comparison of data from the subcohort investigating T2D to (Dayeh et al., 2014) data found 813 consistent sites with 98.5% of sites hypomethylated. Comparing data from both the subcohort investigating T2D and the subcohort investigating HbA1c data to (Volkov et al., 2017) data observed 1,297 (72% hypomethylated) and 712 sites respectively were within differentially methylated regions of the WGBS data. Gene expression: 113 of the 5584 overlapping differentially methylated sites in the prevalence cohort were close to (±10 kb) 65 differentially expressed genes. Incidence study: of the 113 sites identified during gene expression analysis, four sites (within *NKX6.2, SYNPO, RHOT1,* and *CABLES1*) demonstrated reduced risk of T2D (odds ratio <1; p < 0.05).

RR, relative risk; IQR, interquartile range; QC, quality control; SD, standard deviation.

^a^ Only reported results pertaining to T2D. 18 other disease states investigated.

Differential DNA methylation signatures for T2D have been identified in blood samples using microarray platforms in several large population studies, as well as two meta-analyses. In one study investigating the incidence of T2D in people of Indian Asian ancestry (discovery cohort; 1,074 with T2D and 1,590 without T2D) and people of European ancestry (replication cohort; 377 with T2D and 764 without T2D), five CpG sites (*TXNIP, ABCG1, PHOSPHO1, SOCS3,* and *SREBF1*) were identified by Illumina 450 K microarray as having a statistically significant association with T2D after replication (Table 1) (Chambers et al., 2015). While the mechanisms for this variation are unknown, the authors postulate that future functional gene analysis of these genes will reveal involvement in T2D development due to the association of these sites with genes involved in metabolic pathways (Chambers et al., 2015). While in another Illumina 450 K microarray study, 18 CpG loci were significant in a cohort of European ancestry people (563 with T2D and 701 without T2D) and 14 and 16 of these showed the same directional change (p < 0.05) in separate cohorts (Table 1) (Cardona et al., 2019). Gene set enrichment analysis of these sites indicated enrichment in pathways involved in cholesterol biosynthesis, carnitine metabolism and AMPK signalling (Cardona et al., 2019). A more recent study investigated incident (534 with T2D and 13,437 without T2D) and prevalent (348 with T2D and 14,002 without T2D) T2D on a platform screening 752,722 CpG sites within the Generation Scotland cohort (Hillary et al., 2023). This study reported 58 significant CpG sites within the incidence investigation and 52 significant CpG sites within the prevalence investigation, 17 of which overlapped between investigations (Table 1) (Hillary et al., 2023). Here, several significant pathways including cholesterol biosynthesis and cholesterol metabolism were associated with T2D for the prevalence cohort (Hillary et al., 2023). Further, a meta-analysis of European ancestry populations (340 with T2D and 3,428 without T2D; Illumina 450 K Beadchip) revealed 6 CpG sites (*TXNIP, ABCG1, CPT1A, HDAC4, SYNM,* and *MIR23A)* as well as 77 differentially methylated regions that were associated with T2D (Juvinao-Quintero et al., 2021). Analysis on KEGG pathways and GO terms failed to identify any enriched pathways for these genes (Juvinao-Quintero et al., 2021). The authors however caution of correlation with cell-type proportions, with all 6 CpG sites identified associating with white blood cell-type, highlighting the confounding effects of cell-type heterogeneity in blood-based studies (Juvinao-Quintero et al., 2021). This further highlights the need for adjusting for cell-type heterogeneity or cell-type deconvolution in biomarker discovery (Teschendorff and Zheng, 2017; Titus et al., 2017; De Ridder et al., 2024). In a meta-analysis of five prospective studies with people of European descent (1,250 with T2D and 1,950 without T2D; measured on Illumina Methylation EPIC chips or 450 K microarrays: 416,716 - 470,870 probes retained for analysis) 76 CpG sites were associated with T2D after accounting for age, sex, cell-type composition and batch, with four remaining significant after adjustment for BMI (Table 1) (Fraszczyk et al., 2022a). The 76 CpGs were further assessed in a separate replication cohort with people of Indian Asian descent (1,072 with T2D and 1,587 without T2D), where 64 of the CpG sites were directionally consistent (p < 0.05) on a model accounting for age, sex, cell-type composition and batch (Fraszczyk et al., 2022a). Pathway analysis by GO terms and Reactome enrichment analysis revealed several enriched pathways including phospholipid metabolism (Fraszczyk et al., 2022a). While in a smaller cohort (218 with T2D and 77 without T2D) of Indigenous (85% of cohort) and non-Indigenous young people (mean age 15 years with SD ± 3.0 years; 64% female) from Canada, the Illumina EPIC Beadchip was used to identify 3,830 CpG sites (3,725 of which were novel) with ≥1% DNA methylation difference between participants with T2D and those without (Salama et al., 2024). Furthermore, three of these CpG sites, all within the *PFKFB3* gene, were also associated with maternal diabetes exposure during gestation (Salama et al., 2024). These findings indicate that DNA methylation differences between people with T2D and people without T2D are detectable in youth-onset T2D, highlighting the potential for DNA methylation biomarkers unique to this risk group.

The transition to microarray-based screening technology has enabled the identification of a broader range of T2D-associated differential DNA methylation patterns across the genome, far surpassing the capabilities of candidate gene analysis. However, the commonly used microarray platforms such as the Illumina HumanMethylation450 and the MethylationEPIC platforms only enable profiling of ∼413 k and ∼850 k CpG sites respectively (Pidsley et al., 2016), and do not provide base-level resolution of DNA methylation (Figure 1). Whereas, on 10 ng of DNA input, EM-Seq can detect 53.7 million CpG sites and WGBS can detect 36.0 million CpG sites, with 35.8 million CpG sites correlating between detection methods (Vaisvila et al., 2021). Thus, current microarray platforms can, at best, only assess ∼1.5% of the CpG sites that can be profiled by the latest short-read sequencing-based methods.

### 3.4 Sequencing based studies

Whole-genome DNA methylation profiling techniques such as WGBS and EM-Seq far surpass microarray detection techniques due to their unbiased, base-level resolution and the high proportions of CpG sites covered. To the best of our knowledge, there are only three reported applications of whole-genome DNA methylation profiling in the study of T2D, with all utilising case-control designs and WGBS (Table 2). The first study identified 25,820 differentially methylated regions (DMRs) (13,696 hypermethylated and 12,124 hypomethylated) in pancreatic islet cells from six participants with T2D and eight participants without T2D, of which 159 DMRs were assigned to 43 of 65 previously identified candidate genes (Volkov et al., 2017). The second, used WGBS data for a targeted DNA methylation analysis of ± 5 kbs of a CpG site (ch17: 55484635) within the *MSI2* gene in pancreatic islets, revealing 39 differentially methylated positions, 36 of which were hypomethylated (Jeon et al., 2017). This region was selected for investigation after a blood-based 450 K microarray study identified a neighbouring CpG site (chr17:55484600) as differentially methylated and blood-based pyrosequencing within this site confirmed this differential DNA methylation (Jeon et al., 2017). While acknowledging different tissues were screened, the use of WGBS revealed finer-grained detail about the DNA methylation patterns of the *MSI2* gene than either the 450 K microarray or pyrosequencing. The third study, reported 9,025 DMRs (3,269 hypermethylated and 5,756 hypomethylated) mapping to 2,019 differentially methylated genes (DMGs), 77 of which were in previously identified candidate genes, from the spermatozoa from eight men with T2D and nine without (Chen et al., 2020). As demonstrated by these studies, the correlation of multiple DMRs with previously identified candidate genes demonstrates that whole-genome technologies produce concordant findings with earlier DNA methylation detection technologies. Furthermore, the generation of large numbers of DMRs highlights the potential of whole genome technologies to reveal previously unknown relationships between the DNA methylome and T2D, which could reveal new mechanistic insights into the pathogenesis of T2D. Given T2D is a complex and phenotypically heterogeneous condition, future T2D biomarker discovery studies should ideally consider whole-genome profiling techniques to enable a larger proportion of the methylome to be profiled, as many genomic regions of potential change are not assessable by microarray platforms.

**TABLE 2:** Features of Type-2 diabetes studies that have used whole genome sequencing methods to measure DNA methylation.

Reference	Study size	Population summary	Study design	Main results summary
Volkov et al. (2017)	n = 6 with T2D (50% female) n = 8 without T2D (50% female)	Nordic Network for Islet Transplantation donors (Age range not described)	Case-control using WGBS data from pancreatic islets. Validation was by Infinium 450 K microarray (on same cohort) and pyrosequencing (independent cohort [n = 19 with T2D, n = 56 without T2D]). Reads mapped to hg38.	A mean 74% of reads uniquely mapped; 75.9% average methylation level; methylation levels highest in introns (78.5%) and exons (77.4%), and lowest in first exon (34.7%), TSS 200 (25.4%) and TSS 1500 (44.4%); People with T2D had 25,820 DMRs with 13,696 hypermethylated and 12,124 hypomethylated; DMRs with highest methylation difference observed were in *ARX* and *TFAM* genes; 159 DMRs annotated to 43 T2D candidate genes
Jeon et al. (2017)	n = 2 with T2D (100% male) n = 16 without T2D (37.5% male)	People undergoing pancreatectomy at Asan Medical Centers in Seoul, Korea (Age range not described, but mean was 55 ± 16 years)	Case-control validation using WGBS data from pancreatic islets of a study of hyperglycaemia and T2D in a larger cohort screening blood for differential methylation (see Table 2 for details). DNA methylation on and ± 5 kbs from the *MSI2* gene site ch17: 55484635 was investigated. Reads mapped to hg19.	39 statistically significant DMPs were identified between all participants with T2D and participants without T2D (36 hypomethylated). When analysis was limited to males only 32 DMPs were significant between participants with T2D and participants without T2D
Chen et al. (2020)	n = 8 with T2D (100% male) n = 9 without T2D (100% male)	Population not described (20–45 years)	Case-control using WGBS data from spermatozoa. Reads mapped to hg19	87.33%–90.70% genome mapped; assessed methylation across CpG, CHH and CHG (H = A, G or T) and found 9,025 DMRs with 3,269 hypermethylated and 5,756 hypomethylated; 2,019 DMG identified with 77 annotating to previously identified candidate genes (top 10: *IRS1, PRKCE, FTO, PPARGC1A, KCNQ1, ATP10A, GHR, CREB1, PRKAR1A* and *HNF1B*)

TSS, transcription start site; DMR, differentially methylated regions; DMG, differentially methylated genes; DMPs, differentially methylated positions.

### 3.5 The majority of reproducible DNA methylation signatures for T2D have been identified from blood samples

Blood is the most commonly assayed tissue in DNA methylation screening, particularly within studies of large populations. Blood samples are ideal for biomarker development because they are less invasive to collect than the tissues of direct clinical significance, and phlebotomy is already a routine clinical procedure. Importantly, when comparing differential DNA methylation findings for T2D from blood and tissue samples of significance to T2D from the same individual, several CpG marks have been shown to correlate between blood and tissue samples. For example, paired blood and liver samples from 175 people were used as a validation assessment for five blood-identified differentially methylated CpG sites (*TXNIP* [cg19693,031]*, ABCG1* [cg06500161], *PHOSPHO1* [cg02650017], *SOCS3* [cg18181703] and *SREBF1* [cg11024682]), with *TXNIP* (p = 0.02) and *SOCS3* (p = 5.3 × 10^−5^) loci identified to correlate (Chambers et al., 2015). These findings were replicated using matched blood, skeletal tissue and adipose tissue from nine monozygotic twin pairs discordant for T2D, with correlations found between blood and adipose tissue for *SOCS3* and *SREBF1* sites (Regression coefficient = 0.31 and 0.40, p = 0.010 and 0.052, n = 28) (Dayeh et al., 2016). Despite low sample numbers in matched analysis, several studies assaying blood and other tissues in unmatched samples have demonstrated that some tissue-based DNA methylation patterns are reflected in blood. For example, 57.7% of age-related epigenetic changes identified in pancreatic islet cells from 87 participants without T2D were reflected in the blood of a second cohort of 421 participants without T2D (Bacos et al., 2016). Additionally, 67.8% of age-related epigenetic changes in livers of 95 people (35 of which with T2D, but sub-analysis by T2D status was not presented) undergoing Roux-en-Y gastric bypass were also identified in white blood cells of a separate cohort of 421 participants (13,022/13,631 were also concordant with DNA methylation direction) (Bysani et al., 2017). While biomarkers from tissues directly involved in T2D pathology would be ideal, blood appears to be a suitable proxy given its practicalities and the emerging evidence of correlations with tissues of significance. Further studies with matched tissue and blood samples that investigate the extent of the DNA methylation correlation between blood cells and the tissues involved with T2D would further aid in identifying accurate tissue correlated proxy loci that can be assessed by blood cell profiling.

### 3.6 Reproducibility and functions of differentially methylated genes

Several genes associated with differential DNA methylation sites consistent with T2D have been identified by multiple studies and systematic reviews containing case-control data (Table 1) (Muka et al., 2016; Walaszczyk et al., 2018; Willmer et al., 2018). Genes with frequently documented differential DNA methylation in blood samples include *TXNIP, ABCG1, SREBF1* and *CPT1A* in the context of both incidence and prevalence studies. When searching the GWAS catalogue (Sollis et al., 2023), the genes *ABCG1* (Mansour Aly et al., 2021) and *SREBF1* (Ray and Chatterjee, 2020; Vujkovic et al., 2020) have been identified as being significantly associated with T2D. This provides independent lines of evidence that epigenetic changes linked with T2D at these loci are occurring in regions of the genome known to be associated with T2D genetic risk.

When reviewing the biological pathways and molecular functions of these genes, there is either a direct link with cellular mechanisms involved with T2D pathogenesis, or associated cardiometabolic phenotypes. The *TXNIP* gene, which codes for the Thioredoxin-interacting protein, is involved in several inflammatory and redox biochemical pathways, with inflammation as a known byproduct/precipitating factor of T2D development (Choi and Park, 2023). Of particular interest, a murine model has demonstrated that *TXNIP* causes apoptosis in mouse pancreatic *β*-cells when upregulated in response to high glucose levels (Chen et al., 2008). Moreover, the expression of the *ABCG1* gene has been shown in mice to be integral to cellular efflux of cholesterol and prevention of atherosclerosis, a common comorbidity of T2D (Wang et al., 2004; Kennedy et al., 2005; Out et al., 2007). Thus, it has been proposed that increasing *ABCG1* expression may have protective effects against atherosclerosis development (Frambach et al., 2020; Matsuo, 2022). With respect to *SREBF1* (also known as *SREBP1),* a murine model engineered to not express *SREBP1* showed that in the refeed state following a fast, mice without *SREBP1* failed to induce liver lipogenesis (Shimano et al., 1999), an important biochemical pathway for converting excess carbohydrate to lipids. Similarly, the *CPT1A* gene involved in fatty acid oxidation in the mitochondria (Schlaepfer and Joshi, 2020), has been observed to complex with acyl-CoA synthetase and voltage-dependent anion channels in the mitochondrial outer membrane to facilitate uptake of fatty acids (Lee et al., 2011), which is important for maintaining metabolic equilibrium. Whilst detecting correlations of blood-based DNA methylation measures with T2D does not imply the same epigenetic differences exist in the tissue or organs underpinning the condition, such as the pancreas, kidney, and muscle glucose uptake, the direct implication of these genes with strong T2D relevant pathways does indicate that blood cell DNA methylation patterns may reflect changes at the tissue level, or wider systemic epigenetic change as a result of the condition.

## 4 The potential to incorporate epigenetic age for biomarker discovery

Age is a risk factor for many chronic conditions, including T2D, and can be defined as the deterioration of cellular processes that contribute to aberrant health outcomes (López-Otín et al., 2013). A person’s rate of ageing is variable, and can be influenced by multiple internal (intrinsic) and environmental (extrinsic) factors. Epigenomic alterations have been classified as one of 12 hallmarks of ageing, with DNA methylation changes observed to correlate with advancing age (López-Otín et al., 2023). This observation has led to the development of ‘epigenetic clocks’, which utilise machine learning models and regression statistics to estimate the epigenetic age of an individual based on methylation levels at specific CpG sites (Horvath and Raj, 2018). The calculated epigenetic age can then be compared to years since birth age (chronological age) to determine if epigenetic ageing is in acceleration or deceleration (Horvath and Raj, 2018).

While multiple epigenetic clocks have been developed, they can be broadly classified into three generations. First generation clocks use chronological age metrics as the primary source of data in their calibration, with Horvath’s (Horvath, 2013) and Hannum’s (Hannum et al., 2013) clocks the most popular from this generation. Second generation clocks include measures of health and lifestyle factors (e.g., smoking) in their design to better predict morbidity and mortality, including the GrimAge (Lu et al., 2019) and PhenoAge (Levine et al., 2018) clocks. Third generation clocks have transitioned to investigating rates of ageing, which include the DunedinPoAm (Belsky et al., 2020) and its updated version the DunedinPACE clock (Belsky et al., 2022). Of note, all six clocks have been developed for microarray data, highlighting the wide application of microarray technology in biomarker investigation. In addition to this, with the exception of Horvath’s, which used data from 51 healthy tissues, blood was the tissue utilised in development; further highlighting the utility of blood as a suitable tissue for developing epigenetic age estimates.

In addition to being considered as a condition of interest in the development of several clocks (PhenoAge, GrimAge), several studies have investigated the utility of epigenetic age in predicting T2D. For instance, HorvathAge, HannumAge, PhenoAge, GrimAge, Telomere Length, and DunedinPoAm clocks were tested on multiple morbidity and mortality conditions within the Generation Scotland cohort, and several were found to significantly associate with incident T2D (GrimAge Hazard Ratio (HR) = 1.52, 95% CI = 1.20-1.90; PhenoAge HR = 1.54, 95% CI = 1.21–1.97) (Hillary et al., 2020). However, in an investigation with Horvath, Hannum, GrimAge and PhenoAge clocks in a nested case-control T2D study with people from the prospective Doetinchem Cohort Study in the Netherlands, no significant difference was observed with epigenetic ages, and when assessing age acceleration, only the Horvath clock was significant (Fraszczyk et al., 2022b). An investigation of PAI-1, Telomere Length, DunedinPACE, PCHorvath1, PCHorvath2, PCHannum, PCPhenoAge and PCGrimAge clocks was undertaken with participants with and without diabetes from the Swedish Adoption/Twin Study of Aging (study did not distinguish type of diabetes, but assumed 94% of participants had T2D), where it was observed that on smoothened average curves within the 60–70 years age range, DunedinPACE and PAI-1 measures for the participants with diabetes were significantly higher (Wikström Shemer et al., 2024). However when all clocks were assessed by Cox Proportional Hazard modelling no significance was observed (Wikström Shemer et al., 2024). While an investigation of the relationship between DNA methylation age and mortality in a cohort with T2D from Italy found that, after adjusting for risk factors, accelerated epigenetic age was associated with increased mortality (PhenoAge HR = 1.16, 95% CI 1.05-1.28 and DunedinPoAm HR = 3.65, 95% CI 1.43–9.35) (Sabbatinelli et al., 2024). Although it is promising that several epigenetic clocks have observed correlations between ageing rate and T2D, non-significant results with several clocks suggests further refinement of epigenetic clocks for the study of T2D risk is required. One suggested approach for improving the utility of epigenetic age measurements for T2D would be to generate T2D-specific biomarker clocks (Wikström Shemer et al., 2024). Given the above described epigenetic clocks rely on microarray data, a promising method for the identification of novel CpG sites suitable for a T2D specific biomarker is whole genome DNA methylation profiling, with several techniques capable of screening ∼96% of CpG sites. Given that microarrays assess less than one million CpG sites, epigenetic clocks generated from whole genome DNA methylation profiling data have the potential to have greater applicability for T2D prediction and monitoring.

## 5 Future directions

In order to ensure equity in T2D biomarker development, researchers should seek to diversify their study populations to ensure that the translation of biomarkers to the clinic provides equitable representation for populations of a broader set of ancestral backgrounds. The majority of T2D biomarker discovery has occurred within people of European ancestry, biassing findings toward genetic and environmental factors present with people of European origins. It has been demonstrated that the precision of polygenic risk scores for common conditions decays when applied to different ancestry groups (Kachuri et al., 2023; Moreno-Grau et al., 2024). However, the addition of DNA methylation data could assist with improving transferability of polygenic risk scores (McCartney et al., 2018), with some preliminary evidence indicating reduced impact of ancestry on DNA methylation association with medically-relevant phenotypes (Thompson et al., 2022). Whilst it is unclear if the addition of DNA methylation data will improve polygenic risk prediction for people of non-European ancestry, broadening the population diversity in biomarker development will likely have the greatest impact in improving predictive accuracy across diverse ancestries. This approach could mitigate current barriers to diagnosis and management of chronic conditions, ensuring that epigenetic biomarker development delivers more reliable health predictions for underrepresented populations. This need for greater diversity within DNA methylation biomarker development has been demonstrated by a comparison of microarray generated DNA methylation data from blood of healthy people from European ancestry and South Asian ancestry, which observed 16,433 differentially methylated sites; the majority (76%) of which could be ascribed to different cell-type compositions within ancestry groups (Elliott et al., 2022).

T2D is a condition of global significance that disproportionately affects Indigenous Peoples who are impacted by settler colonialism (Harris et al., 2017). While it is encouraging to see studies such as the Strong Heart Study among American Indian communities (Domingo-Relloso et al., 2022), and the iCARE study working with young First Nations people in Canada (Salama et al., 2024), it is imperative further efforts ensure more Indigenous communities have equal opportunity to benefit from biomarker development. Of note, the iCARE study consisted of 85% Indigenous participants (n = 251) and 15% non-Indigenous participants (n = 44), including a subanalysis comparing data between Indigenous and non-Indigenous participants and observed that five of the 3,830 identified differentially methylated CpG sites had differing directions of methylation (Salama et al., 2024). While the high concordance between differential DNA methylation findings between the Indigenous subset and the wider cohort demonstrates the robustness of DNA methylation as a biomarker, the observation of discordant sites suggests a need for greater representation of Indigenous Peoples within T2D biomarker discovery. Furthermore, while not in the context of T2D, a study investigating epigenetic ageing of Native Hawaiian, Japanese American and White participants from Hawaii observed Native Hawaiian people to have a significantly accelerated ageing rate comparative to the other participants (Maunakea et al., 2024). The authors identified several socio-economic and health factors that were protective, as well as several that increased risk, highlighting the importance of the environment in understanding DNA methylation (Maunakea et al., 2024). Given the great sociological and cultural diversity that exists amongst Indigenous Peoples globally, different Indigenous communities likely have different environmental influences that could impact DNA methylation. This highlights the importance of ethical inclusion and equitable representation of Indigenous communities within DNA methylation biomarker research to ensure T2D biomarker development contributes to equitable health outcomes.

Future DNA methylation studies should aim to capture a wide range of age groups, as many prior T2D DNA methylation studies are composed of individuals who are middle aged or older (Tables 1 and 2). Given that DNA methylation patterns change throughout life (Jones et al., 2015), a skew in T2D biomarker studies towards older people risks T2D epigenetic biomarker development becoming further biassed towards older populations. This is of concern for younger people at risk of T2D due to increasing global incidence of younger onset T2D which is often accompanied by more severe phenotypes (Bjornstad et al., 2023). In light of this, future DNA methylation investigations should endeavour to recruit a wide range of age groups to ensure T2D DNA methylation biomarkers enable accurate prediction of condition risk for younger populations.

## 6 Ensuring ethical genomics and equity for Indigenous Peoples in T2D biomarker development

The application of genomics for human health over the past decades has not been equitable, as biases in studies have arguably limited the benefits to specific populations, often to the disadvantage of Indigenous Peoples (Mills and Rahal, 2019; Fatumo et al., 2022). While it could be argued that providing equitable benefits to Indigenous people can be achieved by diversifying recruitment (see discussion below), it could also be argued that true equity can not be achieved while lack of diversity also exists within the human reference genome, genomic functional annotations and databases that catalogue genetic variation. This is of particular importance in the context of T2D biomarker research with Indigenous communities, due to the workflow reliance of popular DNA methylation detection technologies on the human reference genome and functional annotation databases. Currently, the most commonly used human reference genome is GRCh38, which derives ∼70% of its sequence from a single individual of African/European ancestry (Schneider et al., 2017). While recent efforts to improve the human reference genome has seen the release of T2T-CHM13, which improves GRCh38 by filling heterochromatin gaps, the cell lineage utilised is still of majority European ancestry (Nurk et al., 2022). While it is encouraging that improvements to the human reference genome are occurring (Liao et al., 2023), lack of diversity can introduce unknown biases into epigenetic biomarker development. For example, recent whole-genome sequencing of genomes from Indigenous Australians found that while T2T-Ch13 was a more accurate reference than GRCh38, large numbers of unannotated structural variations were identified (Reis et al., 2023). Moreover, 3.4 million single nucleotide variants identified among Indigenous Australians are not present in either the 1,000 Genomes or Human Genome Diversity projects (Silcocks et al., 2023). The lack of diversity in current reference genomes could impact the accuracy of DNA methylation quantification as the human reference genome is typically required as a reference for sequence read mapping and with microarray probe design. If any discordance surrounding CpG loci differences between populations are not accounted for, it is plausible that results may have population-specific biases. Until human reference genomes and analysis methods are able to reflect global human diversity, there is a risk that any T2D DNA methylation biomarkers developed without Indigenous populations may have lower efficacy for the populations with some of the highest burden of T2D.

Rates of T2D in Indigenous communities impacted by colonisation often exceed that of non-Indigenous people of the same country or region (Harris et al., 2017). While the interactions between individual Indigenous communities and the colonial powers (and their established medical and research institutions) on their lands may be unique, many burdens resulting from colonialism are shared. These include, racism, trauma, disruptions to family structures, and shifts towards a Western diet and lifestyle (Wolfe, 2006; Glenn, 2015; Paradies, 2016). Additionally, many Indigenous communities have experienced exploitation of their bodies, bioresources and knowledge by research practices that sought to document, catalogue and exploit Indigenous Peoples and their ways of being (Turnbull, 2007; Reid et al., 2019). In addition to experiencing disproportionate rates of T2D, Indigenous people can experience disadvantages that can arise due to epistemic racism and a failure to consider historical contexts surrounding how Indigenous people have experienced Western healthcare (Sinclaire et al., 2023). For many Indigenous communities, these experiences can carry through generations as intergenerational trauma and have profound effects on health and wellbeing (Griffiths et al., 2016). In this domain, there are concerns that researchers investigating epigenomics of intergenerational trauma with Indigenous communities impacted by colonisation may unintentionally further perpetuate trauma (Saulnier et al., 2022). Thus, it is important that when undertaking genomics studies with Indigenous communities impacted by colonisation, researchers develop an understanding of a community’s individual experiences with colonialism, and how that can impact both their health as well as their relationship with biomedical research.

Attempting to increase diversity in genomics-based biomarker development requires an understanding of what has led to the current lack of diversity. Underrepresentation of Indigenous Peoples within genomics datasets can be partially attributed to self-exclusion due to previous experiences with exploitative Western research practices, lack of consultation with Indigenous Peoples in the design of projects, and the requirements for open-access data (Sherwood, 2013; Garrison et al., 2019; Hudson et al., 2020; Mc Cartney et al., 2022). Sharing data and open access data under the ethos of the Findable, Accessible, Interoperable and Reusable (FAIR) data principles (Wilkinson et al., 2016) is well established in human genomic research. However, open access data is of concern for many Indigenous communities (Hudson et al., 2020). Indigenous Peoples may not receive the same benefits from the use of their genomics data, as communities have raised concerns that they may not have control over who uses their data and what it is used for, with the risk of data being used in ways contrary to cultural protocols (Hudson et al., 2020; Mc Cartney et al., 2022). To mitigate risks from open access data, the Collective benefit, Authority to control, Responsibility and Ethics (CARE) principles were developed (Carroll et al., 2020). The CARE Principles are designed to complement the FAIR principles by providing a framework for Indigenous people to self-determine research objectives and have governance over studies and their associated data (Carroll et al., 2020). If Indigenous Peoples have no control over their data, then the scientific community risks perpetuating the same situations that lead many Indigenous communities to disengage from research in the first place, thereby jeopardising equitable T2D biomarker development. This is critically important if future T2D biomarkers are to be developed with the populations most severely impacted by the condition.

Extensive qualitative work undertaken with Australian Indigenous communities has demonstrated that respectful and ongoing consultation is critical to rebuilding trust in the field (Hermes et al., 2021). In addition to reconciling historical damages, researchers should strive to work in equal partnership with Indigenous Peoples to ensure their research is respectful of Indigenous data sovereignty and governance (Garrison et al., 2019). Researchers and institutes can achieve this through respecting and including Indigenous Knowledge, working in partnership with Indigenous Peoples throughout the project lifecourse (Claw et al., 2018), abiding by CARE principles for data management (Carroll et al., 2020), and developing policies and procedures that are protective of Indigenous biosamples and intellectual property (Garrison et al., 2019). A mechanism through which this can be operationalised is by the creation of project Indigenous governance committees (Hudson et al., 2020) which oversee study operations, sample and data use, and reporting. By empowering Indigenous communities to have authority and ownership over genomics research conducted with them, researchers and institutes can begin rebuilding trust in the genomics field (Hudson et al., 2020). As demonstrated by work undertaken with Indigenous communities in Chile (Arango-Isaza et al., 2023), engagement with Indigenous communities during data interpretation not only returns results to the community and is respectful, but it also provides a richness to the findings that only those with lived experiences can deliver. Finally, when completing the research cycle with reporting, it is important to consider a ‘strengths-based’ as opposed to ‘deficit’ narrative in reporting (Hyett et al., 2019). The above-listed references are a selection of the resources available to the genomics community for enacting Indigenous data sovereignty, for more information see Supplementary Table 1 of Mc Cartney et al. (2022).

There is an urgent need for the research community to build trust by ensuring ethical community engagement, and the inclusion of and governance by Indigenous Peoples, their knowledge, and sovereign rights. Indigenous communities globally are rich and diverse in culture and community protocols, and we provide the following general advice for commencing engagement and partnership with Indigenous communities. We advise learning more about the history and culture of the Peoples you wish to partner with, for example, by undertaking cultural awareness training delivered by an Elder, or community leader. Echoing the advice of Hudson et al. (2020), we recommend seeking out and following local guidelines (co-created with local Indigenous Peoples) for how to respectfully undertake research in partnership with local Indigenous communities. For example, the work we currently undertake is guided by *The South Australian Aboriginal Health Research Accord* (Morey et al., 2023). Inline with local guideline documents, we recommend initiating discussions with community representatives or spokespeople and discuss what expertise and resources the research group has and whether there is a project the community would like to partner on. If the community wishes to co-develop a project, consider collaborating with community members in the project’s design and governance, adhere to CARE principles, and implement local Indigenous data sovereignty procedures throughout the project life cycle (Claw et al., 2018; Carroll et al., 2020; Hudson et al., 2020; Griffiths et al., 2021). Furthermore, it is important for research teams and institutes to support the learning of Indigenous Peoples in how genomics data is generated, managed and analysed (Hudson et al., 2020; Waanders et al., 2023). By enhancing the genomics knowledge of Indigenous Peoples, communities will have a greater capacity to be involved with research, which will contribute toward building trust within the genomics field; thus facilitating better access to the benefits of genomics for Indigenous communities. Without such a considered approach, the T2D health disparity gap experienced by many Indigenous Peoples is at great risk of increasing.

## 7 Challenges of developing DNA methylation biomarkers

### 7.1 Reproducibility challenges

The identification of multiple differential DNA methylation patterns prior to a diagnosis of T2D, as well as post-T2D diagnosis, demonstrates the promise of DNA methylation as a potential biomarker for the identification and monitoring of T2D. However, one major limitation is that few differential DNA methylation patterns have been replicated in more than one cohort. For example, in an examination of seven EWAS studies, only 6.5% (12/185) of differentially methylated sites were identified in more than two studies (Hillary et al., 2023). There are multiple possible explanations for this, including: differing statistical methodologies; different sample sizes and study designs; variation in selection of features corrected for (e.g., age, sex and BMI); definition of T2D (self report versus clinically identified); different technologies used to assess DNA methylation; and variations in population genetics and ancestry (Hillary et al., 2023). Awareness and where possible, controlling for these variations, will improve replicability.

As DNA methylation datasets grow in depth and complexity, the analytical and statistical demands increase accordingly. Researchers in the field of epigenomics have increasingly employed machine learning (ML) strategies (Rauschert et al., 2020), however, several challenges can exist with ML. These include input variable selection, the assessment of model performance, data leakage, model performance metrics, how generalisable the developed model is to the population of interest, and sufficient sample size for analysis (Yousefi et al., 2022). In addition, the way the data is distributed between training, test and prediction data sets; how dependencies are managed within it; confounding variables; data leakage within the analysis pipeline; and balancing of the classes between training, test and prediction dataset, can all influence replicability of ML findings (Whalen et al., 2022). For extensive discussion and best practice mitigation advice on the above listed challenges, we refer the reader to the comprehensive reviews of Whalen et al. (2022) and Yousefi et al., 2022. While ML is delivering detailed findings, DNA methylation is highly variable, nuanced and diverse across both the body and time (Greenberg and Bourc’his, 2019), and ML linear regression based models may not appropriately capture this. To overcome this, a transition towards large language models has been proposed, with recent publication of DNA methylation analytical programs such as MethylGPT (Ying et al., 2024) and CpGPT (de Lima Camillo et al., 2024). These models show great potential due to their network approach, however they have been trained on microarray data, so further development may be required for use with whole genome sequencing data. The increase in dataset complexity combined with the rapid improvements in artificial intelligence facilitated statistics gives great potential to deliver improved biomarkers for T2D prediction and progression.

In addition to statistical considerations, study design could influence replicability. Initial T2D candidate gene studies typically utilised retrospective case-control cohorts, while studies with microarrays used either retrospective case-control cohorts or longitudinal cohorts investigating incident T2D. Many of the above described studies using retrospective case-control designs did not list for participants with T2D, how long they had T2D, or any T2D associated complications. So, for these studies, it is not possible to determine at what stage of T2D progression the described biomarkers correspond to. Furthermore, for the studies investigating incidence of T2D, described DNA methylation differences would be those that presented early in the trajectory of T2D development. Thus, given that T2D is a condition that exhibits a spectrum of clinical presentations (DeFronzo et al., 2015), and that DNA methylation patterns can change in response to health and environmental stimuli (Yousefi et al., 2022), it is possible that studies are identifying DNA methylation patterns that shift as the condition progresses. To improve replicability, future biomarker studies should include, if possible, descriptors of T2D duration and presence/absence of any T2D associated co-conditions to account for the phenotypical heterogeneity that occurs with this condition.

Study sample size can also influence replicability. Many candidate gene T2D DNA methylation studies had small sample sizes (often only in the 10s), which greatly increases the possibility of type II errors. The transition towards microarray technology has been accompanied by larger sample sizes, with hundreds to thousands of samples being common (Table 1). However, a major limitation of microarrays is they are limited to ∼900 k CpGs or less; over an order of magnitude less than the ∼30 million CpG sites possible with methylome sequencing (Vaisvila et al., 2021). Thus, a transition to sequencing based methods, will enable the relationship of DNA methylation with T2D to be studied at genome-wide scale. However, to support this, larger sample sizes will be required to avoid type II errors. As DNA methylation detection technologies improve and become cheaper, more replicates will facilitate further understanding of the relationship between DNA methylation and T2D.

Cell-type heterogeneity can also influence DNA methylation measures as different cell-type feature distinct DNA methylation patterns (Loyfer et al., 2023). As discussed above, blood is the most common tissue in T2D DNA methylation studies, however potential confounding can occur due to blood being composed of multiple different cell-type that can exhibit variation in their proportionality. When investigating DNA methylation from blood, cell-type heterogeneity should be accounted for. One method to control for cell-type composition is fluorescence-activated cell sorting (FACS) (Bonner et al., 1972), however this process requires specialised equipment, can be costly and must occur before DNA extraction. To overcome the infeasibility of FACS in large studies, several computational based methods have been developed to account for cell-type heterogeneity directly from DNA methylation data, with tools such as *EpiDish* performing particularly well for blood with both microarray and methylome sequencing data (Houseman et al., 2012; Teschendorff et al., 2017; De Ridder et al., 2024) This is particularly promising for future T2D biomarker studies, as cell-type variation can be accounted for in analysis and thus bypasses the need for physical sorting of cells prior to analysis.

### 7.2 Practical limitations of whole genome sequencing

While whole genome sequencing based DNA methylation approaches deliver the highest resolution datasets, several challenges present with methylome sequencing. Firstly, comparative to other techniques, sequencing approaches require more specialised equipment and are more costly to generate data. Secondly, the per-sample sequence data are exponentially larger than from candidate gene studies or microarrays, and often require high-performance computing (HPC) for data management and analysis; both of which can be costly (Figure 1). Moreover, the increasingly complex genomics datasets that are being generated require advanced data analysis expertise (Krishna and Elisseev, 2021). In the realm of biomarker development, the complexity of these datasets, combined with the need to use more sophisticated analytical methods introduces new challenges. One such challenge is the ‘curse of dimensionality’, where the large number of features vastly exceeds the sample size, making it difficult to identify meaningful patterns and increasing the risk of overfitting, thereby complicating statistical evaluation across all dimensions. This added complexity, correspondingly increases the cost and resource requirements of methylome sequencing based studies, thus making it more challenging for research teams that have a limited capacity to handle and analyse large volumes of complex sequence data.

### 7.3 Ethical and privacy considerations

Progress in biomarker development towards sequencing-based methylome profiling introduces additional participant privacy considerations. When considering sharing DNA methylation data obtained from sequencing, researchers should be aware that it may be possible to re-identify people and their families, and in the future, potentially deduce other information regarding health and lifestyle (Santaló and Berdasco, 2022). While the level of risk to participant privacy with data sharing is still to be determined, it cannot be ignored, due to the potential harms that can arise from exploitation of this information (Oestreich et al., 2021). There are several strategies that can be adopted to protect participant privacy, including keeping data secured through mechanisms such as restricted access and encryption, and anonymising identifiable information prior to public release (Bonomi et al., 2020). Furthermore, researchers should also be aware that epigenomic data may not receive the same level of legal protection as genomics data, which can have privacy implications, particularly when non-medical industries, such as insurance and forensics, express interest in this data (Dupras et al., 2018). For further discussion on ethico-legal considerations of epigenetic data and consent management, see (Dupras et al., 2019; 2020; Oliva et al., 2024). Given that some populations continue to experience discrimination and prejudice based on aspects of their identity, such as their ethnicity, health status and gender, we advise that the technique selected to protect privacy be informed by consultation with the study population(s). Although focusing on populations comfortable with open access data may offer easier data access and management, investing in the creation of secure data environments for all populations would have wide-ranging benefits.

## 8 Discussion

Our review of the microarray and sequencing-based studies above reveals some common biological pathways and functions associated with differentially methylated CpG sites, particularly in cholesterol biosynthesis, insulin signalling, and metabolism, all of which are linked to the pathogenesis of T2D and its associated complications. However, it is important to acknowledge that there are significant challenges in linking differentially methylated CpGs to precise biological functions. Firstly, CpG sites are often associated with nearby genes based solely on linear genome proximity, which may fail to reflect the complex regulatory interactions between DNA methylation at distal regulatory elements as well as gene expression (Taher and Ovcharenko, 2009; McLean et al., 2010; Yao et al., 2015). Secondly, many studies focus on DNA methylation in white blood cells, which, while easily accessible via phlebotomy, are not the primary tissue involved in T2D pathogenesis. As a result, inferring gene regulatory changes in key tissues, such as the pancreas or liver, is problematic, and it is unrealistic to expect robust causal mechanistic insights from these data alone. Nonetheless, in the case of blood-based studies, it is plausible that T2D could induce epigenetic changes in white blood cells through systemic effects driven by T2D, which could explain the observed DNA methylation changes. While blood based studies exhibit these limitations, blood based biomarkers will likely have the greatest clinical translation potential, due to the comparative ease of phlebotomy over tissue biopsy. Moreover, the systemic nature of T2D and its complications may increase stochastic variation in the epigenome, potentially explaining why different studies detect varying epigenetic changes. From a biomarker development perspective, whilst linking any changes in DNA methylation to mechanisms linked with T2D pathogenesis would increase confidence in the results, it is important to recognise that robust biomarkers may still be developed based on consistent DNA methylation patterns, even without direct mechanistic insights, provided they reliably associate with health condition states or outcomes across independent cohorts. These biomarkers could offer valuable predictive or diagnostic potential, especially when used in conjunction with other molecular and clinical data.

We have highlighted the importance of ensuring genomic technologies are equitably and ethically accessible for Indigenous Peoples. This is of particular importance given the discussed potential for predictive biomarkers from DNA methylation data for T2D; a condition with disproportionate prevalence within many Indigenous communities (Harris et al., 2017). Our review highlights how genomics research poses barriers for Indigenous Peoples and offers guidance on how researchers can adapt their approaches to uphold the highest ethical standards when collaborating with Indigenous communities. We have attempted to discuss all these issues from a global perspective and to provide general Indigenous data sovereignty advice that can be used by all, but we acknowledge that we write from the perspective of Aboriginal (SM and AB) and non-Aboriginal (SB) Australians whose research work is predominantly in partnership with Australian Indigenous communities. We acknowledge the rich diversity that exists within Indigenous communities and appreciate that aspects of our advice may not apply in all contexts. Regardless, we implore that researchers who wish to undertake genomics work with Indigenous communities form genuine collaborations with Indigenous Peoples and be led by the directives of the communities they partner with.

In summary, based on the study sizes reviewed herein, the complex nature of T2D pathogenesis, and the observation that many Indigenous populations globally exhibit the highest prevalence rates, there are key limitations in previous biomarker studies, particularly the insufficient genome-wide coverage of microarray platforms. To advance T2D biomarker development, we see good evidence that future efforts should focus on: 1) large longitudinal cohorts to increase statistical power, 2) the inclusion of diverse populations, particularly Indigenous populations with higher T2D prevalence, and 3) sequencing-based DNA methylation profiling at the discovery stage, which allows for the incorporation of diverse genetic backgrounds and enables broader genome-wide coverage in analyses. By embracing these strategies, the field can move closer to developing robust, globally relevant biomarkers that not only advance our understanding of T2D but also pave the way for more equitable healthcare solutions for those most affected by this complex condition.
